# Patient-derived cell models as preclinical tools for genome-directed targeted therapy

**DOI:** 10.18632/oncotarget.4627

**Published:** 2015-07-16

**Authors:** Ji Yun Lee, Sun Young Kim, Charny Park, Nayoung K.D. Kim, Jiryeon Jang, Kyunghee Park, Jun Ho Yi, Mineui Hong, Taejin Ahn, Oliver Rath, Julia Schueler, Seung Tae Kim, In-Gu Do, Sujin Lee, Se Hoon Park, Yong Ick Ji, Dukwhan Kim, Joon Oh Park, Young Suk Park, Won Ki Kang, Kyoung-Mee Kim, Woong-Yang Park, Ho Yeong Lim, Jeeyun Lee

**Affiliations:** ^1^ Division of Hematology-Oncology, Department of Medicine, Samsung Medical Center, Sungkyunkwan University School of Medicine, Seoul, Korea; ^2^ Samsung Genome Institute, Samsung Medical Center, Seoul, Korea; ^3^ Innovative Cancer Medicine Institute, Samsung Cancer Center, Samsung Medical Center, Seoul, Korea; ^4^ Department of Pathology and Translational Genomics, Samsung Medical Center, Sungkyunkwan University School of Medicine, Seoul, Korea; ^5^ Oncotest, Freiburg, Germany; ^6^ Department of Molecular Cell Biology, Sungkyunkwan University School of Medicine, Seoul, Korea; ^7^ Division of Hematology-Oncology, Department of Medicine, Hanyang University Hospital, Seoul, Korea

**Keywords:** gastric cancer, patient-derived cells, genomic analysis, targeted therapy

## Abstract

**Background:**

In this study, we established patient-derived tumor cell (PDC) models using tissues collected from patients with metastatic cancer and assessed whether these models could be used as a tool for genome-based cancer treatment.

**Methods:**

PDCs were isolated and cultured from malignant effusions including ascites and pleural fluid. Pathological examination, immunohistochemical analysis, and genomic profiling were performed to compare the histological and genomic features of primary tumors, PDCs. An exploratory gene expression profiling assay was performed to further characterize PDCs.

**Results:**

From January 2012 to May 2013, 176 samples from patients with metastatic cancer were collected. PDC models were successfully established in 130 (73.6%) samples. The median time from specimen collection to passage 1 (P1) was 3 weeks (range, 0.5–4 weeks), while that from P1 to P2 was 2.5 weeks (range, 0.5–5 weeks). Sixteen paired samples of genomic alterations were highly concordant between each primary tumor and progeny PDCs, with an average variant allele frequency (VAF) correlation of 0.878. We compared genomic profiles of the primary tumor (P0), P1 cells, P2 cells, and patient-derived xenografts (PDXs) derived from P2 cells and found that three samples (P0, P1, and P2 cells) were highly correlated (0.99–1.00). Moreover, PDXs showed more than 100 variants, with correlations of only 0.6–0.8 for the other samples. Drug responses of PDCs were reflective of the clinical response to targeted agents in selected patient PDC lines.

**Conclusion(s):**

Our results provided evidence that our PDC model was a promising model for preclinical experiments and closely resembled the patient tumor genome and clinical response.

## INTRODUCTION

With rapid advances in molecular oncology, the availability of preclinical *in vitro* cell models and *in vivo* animal models with specific genomic aberrations is critical for improved prediction of clinical outcomes in cancer patients. One of the most widely used preclinical models is conventional cell lines, such as the NCI-60 panel of cell lines [1]; these cell lines are widely used in preclinical testing for novel targeted drugs, partially owing to the low expense and reduced labor associated with cell culture compared with other preclinical models, such as animal xenografts. However, recent studies have shown that accumulation of genetic aberrations in cancer cell lines occurs with increasing passage number. These models also lack the heterogeneity of tumors and do not exhibit a proper microenvironment, highlighting the limitations of cell-based models [2–5]. Consistent with this, Johnson et al. demonstrated that *in vivo* activities of the cell lines within the NCI-60 panel did not closely correlate with corresponding human cancers [6].

Therefore, to better preserve the genomic integrity and tumor heterogeneity observed in patients, patient-derived xenograft (PDX) models are being used more frequently [7–9]. PDX is generated by directly transplanting freshly resected patient tumors into immunocompromised murine hosts with or without an intermediate *in vitro* culture step [10]. This PDX model is an improvement over cell lines because it can provide both an appropriate tumor microenvironment and heterogeneity of tumor cells. However, the engraftment success rates and growth rates of implanted tumors are highly variable depending on the tumor type, possibly due to insufficient numbers of hematopoietic cells and/or ineffective microenvironmental cues in the mouse stroma [11, 12]. The extent to which tumor cells from freshly resected tumors are able to withstand mechanical stresses and xenotransplantation barriers is also unclear [13]. Furthermore, the use of PDX models for application in clinical oncology is limited owing to the time required for PDX establishment (> 4 months) since most patients with refractory cancer live less than 1 year. Recently, PDC line models have been suggested as an alternative preclinical model [14] to be used as a prediction tool for preclinical drug sensitivity.

Therefore, in this study, we aimed to overcome these potential barriers of pre-existing models by examining the capacity of PDC line models to recapitulate the histological and genomic features of primary patient tumors. In selected cases, we screened drug sensitivity *in vitro* using PDC lines and compared the results with real-life clinical treatment outcomes.

## MATERIALS AND METHODS

### Patient consent and study inclusion

Between April 2012 and August 2014, patients with metastatic cancer were enrolled in the SMC Oncology Biomarker study (NCT#01831609,http://clinicaltrials.gov). Briefly, the inclusion criteria were as follows: age ≥ 18 years; pathologically confirmed solid cancer; presence of metastatic lesion(s) not amenable to surgical treatment and having malignant effusion in the body cavity which needed to be drained by percutaneous methods for therapeutic purpose. Effusions were obtained for therapeutic purposes after obtaining written informed consent, and all procedures were carried out according to guidelines from the Declaration of Helsinki. The Institutional Review Board at the Samsung Medical Center approved the protocol. Of the 200 patients who had given written informed consent, 24 patients retracted their consent during the course of the study. Thus, the processes described below were performed for the remaining 176 patients.

### Primary cultures of human effusions

Malignant ascites, pleural effusions, or pericardial effusions were collected from patients with metastatic cancer. Collected effusions (1–5 L) were divided into 50-mL tubes, centrifuged at 1500 rpm for 10 min, and washed twice with PBS. Cell pellets were resuspended in culture medium and plated into 75-cm^2^ culture flasks. Cells were grown in RPMI 1640 supplemented with 10% fetal bovine serum (FBS; Gibco BRL, Paisley, UK) and 1% antibiotic-anti-mycotic solution (Gibco BRL). The medium was changed every 3 days, and cells were maintained at 37°C in a humidified 5% CO_2_ incubator. PDCs were passaged using TrypLE Express (Gibco BRL) to detach cells when the cells reached 80–90% confluence.

### Cryopreservation of PDCs

Cells at 80–90% confluence were washed, detached using TrypLE Express, and incubated for 3 min at 37°C with 5% CO_2_. Following detachment, 4 mL of complete culture media was added to block trypsin activity, and cells were transferred to a 15-mL sterile centrifuge tube. After centrifugation, cells were resuspended in 1 mL of freezing medium (Cellbanker, Zenoaq, Japan), transferred into cryovials (Nalge Nunc, Naperville, IL, USA), and frozen at −80°C overnight.

### DNA/RNA extractions

Cultured primary human cells (passage 1 to 4, Supplementary Table 1) were harvested with TrypLE Express. Genomic DNA was isolated using a QIAamp DNA Mini Kit (Qiagen, GmBH, Hilden, Germany), and total RNA was isolated with an RNeasy Mini Kit (Qiagen) according to the manufacturer's instructions. The concentrations of genomic DNA and RNA were measured using a NanoDrop ND-100 (Nano Drop Technologies, Wilmington, DE, USA). Genomic DNA and RNA were stored at −80°C.

### Ion AmpliSeq Cancer Panel v2

We used the Ion AmpliSeq Cancer Panel v2 (Life Technologies, USA) to detect common somatic mutations as previously described [15]. This panel was used to assay 2, 855 mutations in 50 commonly mutated oncogenes and tumor suppressor genes (Supplementary Table 2). DNA samples were subjected to single-tube, multiplex polymerase chain reaction (PCR) amplification using the Ion AmpliSeqCancer Primer Pool and Ion AmpliSeqKit reagents (Life Technologies). Treatment of the resulting amplicons with the FuPa Reagent partially digested the primers and phosphorylated the amplicons. The phosphorylated amplicons were ligated to Ion Adapters and purified. For barcoded library preparation, we substituted barcoded adapters from the Ion Xpress Barcode Adapters 1–96 Kit for the non-barcoded adapter mix supplied in the Ion AmpliSeq Library Kit. Ligated DNA was subjected to nick-translation and amplification to complete the linkage between adapters and amplicons and to generate a sufficient material for downstream template preparation. Two rounds of Agencourt AMPure XP Reagent binding at 0.6 and 1.2 bead-to-sample volume ratios were used to remove input DNA and unincorporated primers from the amplicon-containing solution. The final size of the library molecules was approximately 125–300 bp. We transferred the libraries to the Ion OneTouch System for automated template preparation. Sequencing was performed on an Ion PGM sequencer according to the manufacturer's instructions. We used IonTorrent Software for automated data analysis.

A new pipeline was designed for highly sensitive identification of Single nucleotide variations (SNVs) for passages 0, 1, and 2 (P0, P1, and P2, respectively) and xenografts. Varscan2 SNP calling was performed with the following options: min-coverage, 50; min-var-freq, 0.01; and *p*-value, 0.1. Variants around the insertions/deletions (InDels) were filtered out [16]. Variants were annotated using Oncotator [17].

### Targeted sequencing

In order to genomically compare PDCs to primary tumor specimens, we performed targeted deep sequencing in 16 primary tumor-PDC paired samples. Genomic DNA was extracted, and a SureSelect customized kit (Agilent Technologies, Santa Clara, CA, USA) was used for capturing 381 cancer-related genes. Illumina HiSeq 2500 was used for sequencing with 100 bp paired-end reads. The sequencing reads were aligned to the human genome reference sequence (hg19) using BWA-mem (v0.7.5), SAMTOOLS (v0.1.18), Picard (v1.93), and GATK (v3.1.1) for sorting SAM/BAM files, duplicate marking, and local realignment, respectively. Local realignment and base recalibration were carried out based on dbSNP137, Mills indels, HapMap, and Omni. SNVs and InDels were identified using Mutect (v1.1.4) and Pindel (v0.2.4), respectively. ANNOVAR was used to annotate the detected variants. Only variants with over 1% of allele frequency were included in the results. The correlation coefficient was calculated based on variants that were detected in both cells.

### nCounter Copy Number Variation CodeSets

For detection of copy number variations (CNVs), 300 ng of purified genomic DNA extracted from PDCs was analyzed using nCounter Copy Number Variation CodeSets. DNA was fragmented by AluI digestion and denatured at 95°C. Fragmented DNA was hybridized with the codeset of 86 genes in the nCounter Cancer CN Assay Kit (Nanostring Technologies, Seattle, WA, USA) for 18 h at 65°C and processed according to the manufacturer's instructions. An nCounter Digital Analyzer was used to detect and tabulate the signals of the reporter probes. Average count numbers of greater than 3 were called and confirmed by immunohistochemistry (IHC), fluorescent *in situ* hybridization (FISH), or real-time PCR. Validation of nCounter results has been published previously [15].

### NanoString 522-kinase panel

The nCounter GX Human Kinase Kit (NanoString Technologies) was used for targeted gene expression analyses of PDCs. Purified RNA (100 ng) was hybridized with the available 522 gene code set for 18 h at 65°C and processed according to the manufacturer's instructions [18].

### PDX models established from PDCs

PDCs were transferred to OncoTest, Germany as frozen vials. On site, the cells were thawed, the freezing medium was removed, and the cells were resuspended and transferred into T75 flasks. Cells were grown for 3–7 days in RPMI/10% FBS until the culture reached around 80% confluence. Cells were collected and counted, and 5,106 cells were injected into the hind flanks of NOD scid gamma (NSG) mice (Jackson Laboratories). Tumors developed within 25–85 days after injection; these tumors were explanted, and viable portions of the tumors were cut into pieces and implanted subcutaneously into female NMRI nu/nu mice (Harlan Laboratories). This process was repeated in order to serially passage the respective models. From each passage, formalin-fixed, paraffin-embedded (FFPE) blocks were prepared, and tumor slices were stained with hematoxylin and eosin (H&E). Slides were scanned with a Hamamatsu slide scanner, and images were extracted using the Nanozoomer program from Hamamatsu. All animal handling and experiments with animals were in accordance with the guidelines set by the Samsung Biological Research Institute.

### Statistical analysis

Statistical analysis was designed to reduce the potential for false-positive calls from the mutation analysis by the Ion AmpliSeq Cancer Panel v2. The variants were filtered by coverage (>100×), quality score (>30), and variant frequency of each sample (>1%). We also discarded germline variants specific to Koreans, such as rs1042522 in *TP53* and rs1870377 in *KDR*. Nonsynonymous and frame shift mutations were included for further analysis. We used the MyCancerGenome database (http://www.mycancergenome.org/) and Therapeutic Target Database (http://bidd.nus.edu.sg/group/TTD/ttd.asp) to address clinically actionable mutations.

For expression analysis, high-throughput gene expression analyses were carried out for TCGA gastric cancer (GC; *N* = 307), ACRG (*N* = 300), and our PDCs (*N* = 109) [19, 20]. The TCGA STAD dataset was analyzed using gene reads per kilobase per million mapped reads (RPKM) estimated by the TCGA pipeline with the Illumina GA and HiSeq 2000 platforms. ACRG was analyzed using the log10-transformed RMA signal intensity with the Affymetrix Human Genome U133 Plus 2.0 Array. Before integrating multiple datasets, our PDC gene expression data were normalized by nSolver and adjusted for removing outliers using the R package ‘outliers’ (http://cran.r-project.org/web/packages/outliers/index.html). Next, platform effects of the three datasets were eliminated by meta-analysis using ComBat [21]. Finally, a gene expression matrix of 485 genes and 716 samples was used for future analysis.

In order to investigate gene expression concordance in PDCs, we performed correlation analyses between different cohorts, PCA analysis, sample hierarchical clustering (Euclidean distance and average method), and Turkey's honest significant difference test for interesting genes and five groups. All future analysis was performed using R language.

## RESULTS

### Patient characteristics

**Figure 1:**
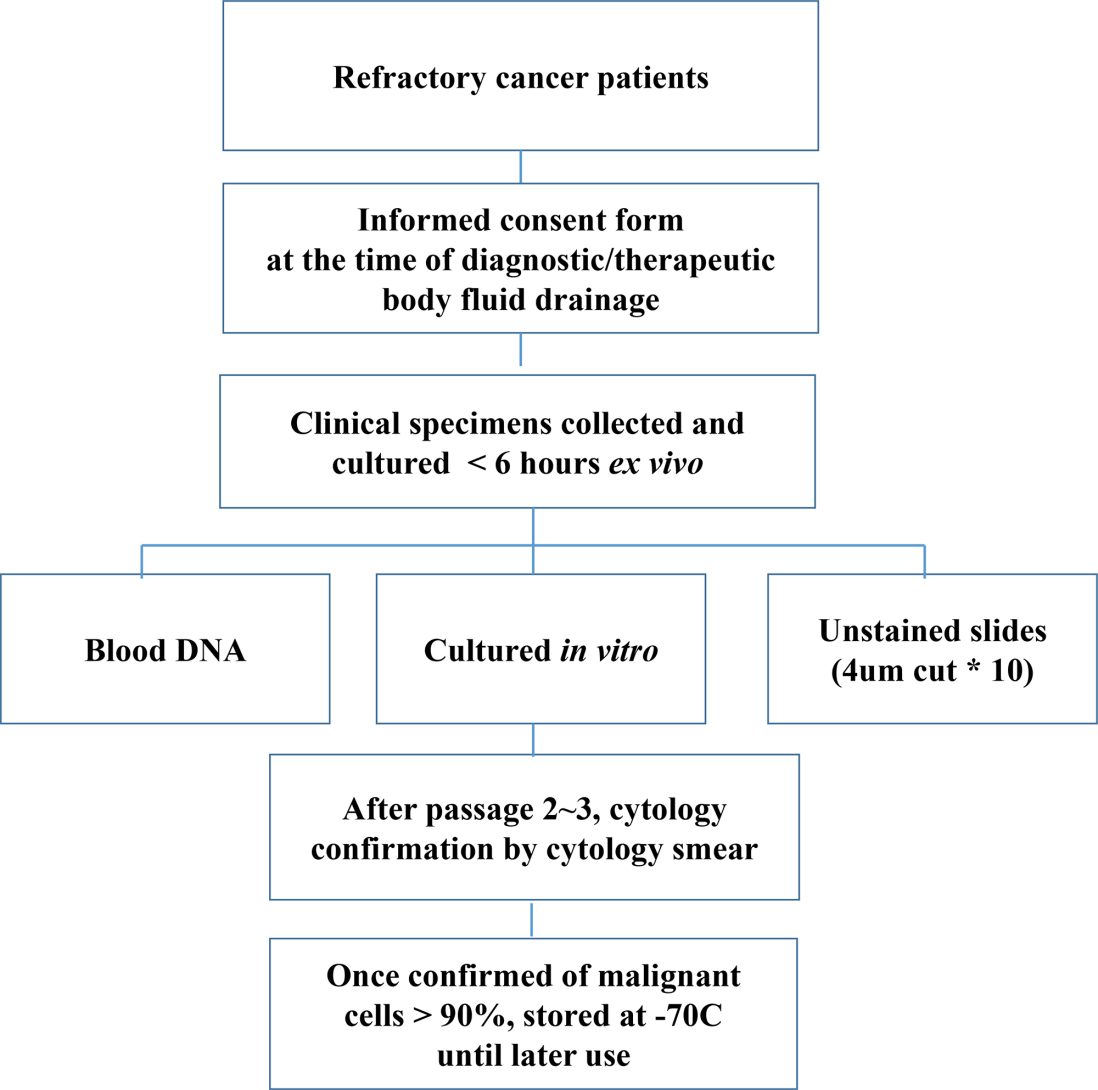
Workflow for establishing PDC models

From January 2012 to May 2013, 176 patient samples were collected for this study, and PDC models were successfully established in 130 (73.6%) of these samples. Successful PDCs were defined as those cells that were cytologically confirmed by a designated pathologist (M.H. or I.D.) and those that maintained growth following two passages. Cells from all patients were stained with either H&E or Papanicolaou, and micrographs were prospectively stored in our internal database. The most common reasons for culture failure were bacterial contamination (26 cases, 56.5%; Figure 1), followed by failure of the cells to grow in culture (16 cases, 34.8%); in 4 cases (8.7%), no cells were found in the collected effusion. Of 130 PDC models, 14 cases that were inadequate for genomic profiling were excluded. Therefore, genomic analyses, including Ion Ampliseq, nCounter CNV Assay, and Nanostring-based targeted gene expression profiling were performed in 116 cases (Supplementary Table 1).

Table 1 summarizes the baseline characteristics of the patients (*N* = 116). The most common cancer type was GC (*N* = 58; 50.0%), followed by colorectal cancer (*N* = 25; 21.6%) and then hepatocellular carcinoma (*N* = 8; 6.9%). PDCs were collected most commonly from ascites (*N* = 101; 87.1%), followed by pleural effusions (*N* = 12; 10.3%), pericardial effusion (*N* = 1; 0.9%), and other sources (Table 1). The maximum passage number of cells was 10 (range, 6–14), and passage numbers at the time of analysis ranged from 1 to 4 (Supplementary Table 1). All PDCs were grown in flasks as attached monolayers. Details of growth properties of established PDCs are described in Supplementary Table 1. With our culture conditions, the median time between primary tumors and P2 for this model was 4 weeks (average 28.6 days). In all cells, the median time from specimen collection to P1 was 3 weeks (range, 0.5–4 weeks), while that for growth from P1 to P2 was 2.5 weeks (range, 0.5–5 weeks). The median doubling time was 119.5 h.

**Table 1:** Baseline patient characteristics (*N* = 116)

Variable	Patients (*N* = 116)	%
Age-year		
Median	55	
Range	21–80	
Sex		
Male	61	52.6
Female	55	47.4
Cancer Types		
Gastric cancer	58	50.0
Colorectal cancer	25	21.6
Hepatocellular carcinoma	8	6.9
Pancreatic cancer	6	5.2
Cholangiocarcinoma	3	2.6
Sarcoma	4	3.4
Non-small cell lung cancer	3	2.6
Neuroendocrine tumor	3	2.6
Melanoma	2	1.7
Renal cell carcinoma	1	0.9
Esophageal squamous cell	1	0.9
Gall bladder cancer	1	0.9
Genitourinary cancer	1	0.9
Source of PDCs		
Ascites	101	87.1
Pleural effusion	12	10.3
Pericardial effusion	1	0.9
Others	2	1.7

### Genomic landscape of the PDCs

**Figure 2:**
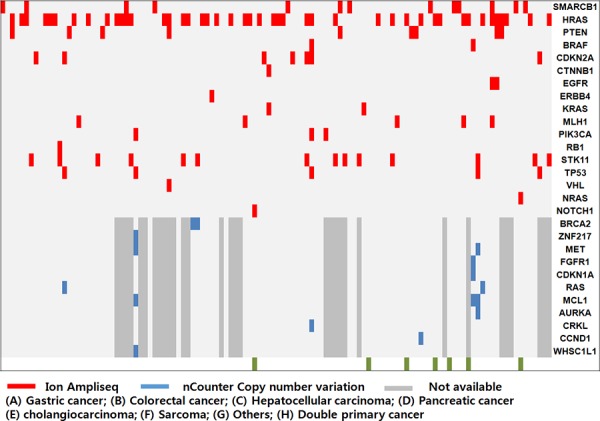
Mutational analysis of the patient-derived cell (PDC) cohort Overall genetic alterations in PDCs were identified by Ion Ampliseq (red) and nCounter Copy number variation assay (blue).

Of the 130 PDCs, we successfully obtained genomic profiling in 116 PDCs. We identified 181 mutations in 50 genes using Ion Ampliseq 2.0; most of these mutations were nonsynonymous point mutations. In addition to the point mutations, one truncation mutation was found in *APC*, and two and one frameshift mutations were found in *TP53* and *VHL*, respectively. The most commonly detected molecular aberrations were found in *HRAS*, *SMARCB1*, *STK11*, *PTEN*, *CDKN2A*, *TP53*, *MLH1*, *PIK3CA*, *BRAF*, *EGFR*, and *KRAS* (Figure 2). We used the MyCancerGenome database (http://www.mycancergenome.org/) and Therapeutic Target Database (http://bidd.nus.edu.sg/group/TTD/ttd.asp) to evaluate clinically actionable mutations. Actionable mutations were identified in 76 of 116 PDCs screened in this study. Using CNV assays, we identified 16 amplifications in 11 genes: three in *MCL1*; two each in *BRACA2*, *MET*, and *RAS*; and one each in *ZNF217*, *FGFR1*, *CDKN1A*, *AURKA*, *CRKL*, *CCND1*, and *WHSC1L1* (Figure 2).

### PDCs were reflective of genomic alterations in parent tumors and clinical phenotypes in response to targeted agents

**Figure 3:**
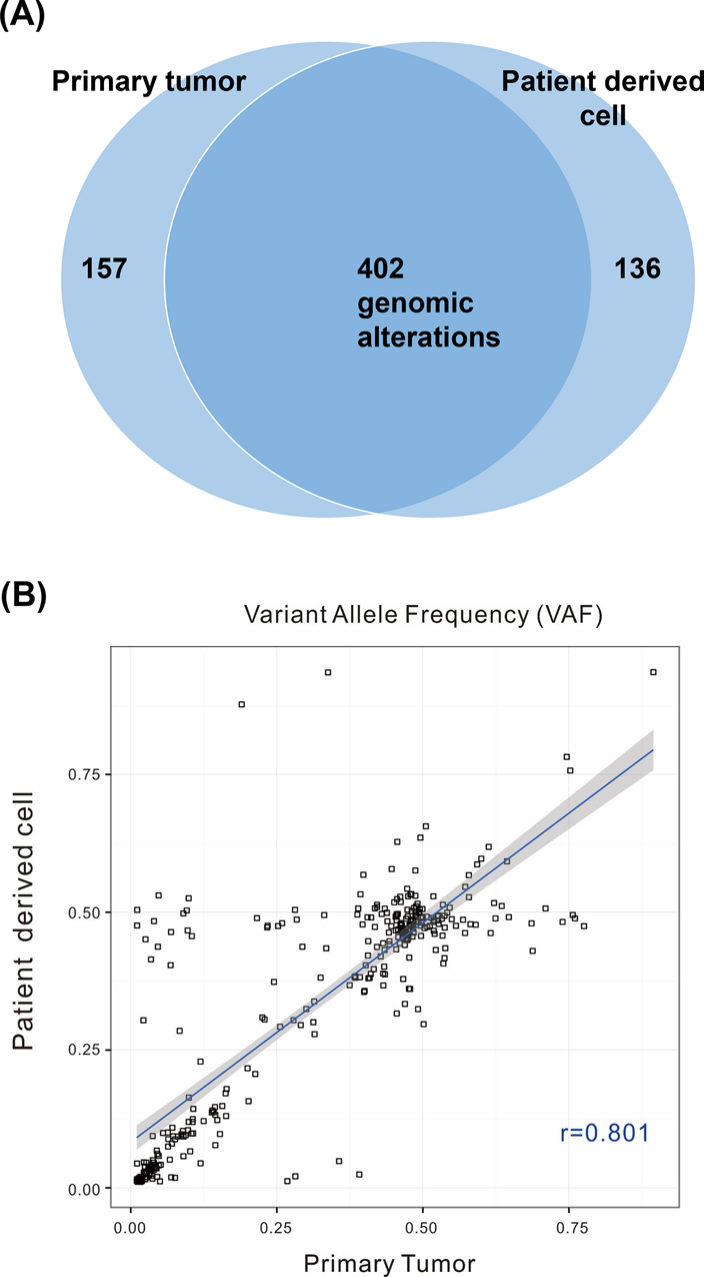
A. Venn diagram showing the variants detected in the primary tumor and patient-derived cells (PDCs) Among 695 genomic alterations from 32 samples, 402 were commonly detected from both types of cells. **B.** Correlations of variant allele frequencies (VAFs) between primary tumor PDCs. The plot shows VAFs of commonly identified SNVs and InDels from 32 samples. The Pearson correlation coefficient between the variants from primary tumor cells and PDCs was 0.801.

Next, we evaluated whether PDCs maintained the genetic and histological features of their parent tumors in 16 pairs of PDCs and primary tumors from synchronous patients with metastatic cancer. Synchronous samples were defined as those collected less than 6 months apart based on the date of the primary tumor procurement. Deep sequencing of 16 paired samples revealed that genomic alterations were highly concordant between primary tumors and the progeny PDCs (Figure 3 and Table 2), with an average VAF correlation of 0.878.

**Table 2:** Genetic correlation of primary tumors and the corresponding patient-derived cells using targeted sequencing

Case #	VAF correlation(>=0.1)	Intersection
S-1	0.978	20
S-2	0.988	26
S-3	0.954	36
S-4	0.961	24
S-5	0.903	27
S-6	0.952	29
S-7	0.969	25
S-8	0.979	28
S-9	0.779	22
S-10	0.802	35
S-11	0.699	33
S-12	0.992	17
S-13	0.855	30
S-14	0.848	4
S-15	0.756	27
S-16	0.633	19
Average/total	0.878	402
Total SD	0.11439697	7.83049594

Next, we examined whether the drug response profiles of PDCs were reflective of the clinical response to targeted agents in selected patients who had such information available. PDC#001 was generated from the sample collected from a patient with GC who was sensitive to lapatinib treatment, which was reflected in the corresponding PDC, with a half-maximal inhibitory concentration (IC_50_) of 1.1 μM (Table 3). PDC#014 was generated from the sample collected from a patient with BRAFV600E (+) melanoma, who was resistant to vemurafenib. The clinical response to vemurafenib (resistance) and sensitivity of PDCs generated from this patient correlated very well, with an IC_50_ of more than 10 μM for vemurafenib. PDC#042 was generated from the sample collected from a patient with HCC, who was resistant to sorafenib and had an IC_50_ for vemurafenib of more than 2.0 μM. PDC#51 and PCD#74 were generated from samples collected before sorafenib treatment in patients with HCC who later showed stable disease after sorafenib treatment for more than 4 months. The IC_50_ values for sorafenib in these two HCC PDCs were below 2.0 μM, although a larger cohort will be needed to define the cut-off value for prediction of the actual clinical response.

**Table 3:** Correlation between drug sensitivity profile and the actual response to targeted agents

PDC#	Diagnosis	Doubling time (hr)	Analysis/stock passage	Maximum passage	Treatment outcome to targeted agents	IC_50_	Highlight genomic alteration	Clinical response
001	Gastric cancer	85.92	2	7	Lapatinib	1.1	SMARCB1, HER2	Sensitive
009	Hepatocellular carcinoma	72	3	9	Sorafenib	2.2		Resistant
011	Hepatocellular carcinoma	116.4	2	7	Sorafenib	2.3	HRAS	Resistant
014	Melanoma	57.6	2	12	Vemurafenib	> 10	BRAF, FGFR1, CDKN1A, MCL1	Resistant
042	Hepatocellular carcinoma	115.6	2	9	Sorafenib	2.1		Resistant
045	Hepatocellular carcinoma	65.02	3	13	Sorafenib	2.1	HRAS, STK11	Resistant
051	Hepatocellular carcinoma	57.8	2	12	Sorafenib	1.6	MLH1	Sensitive
076	Hepatocellular carcinoma	57.8	3	11	Sorafenib	1.5		Sensitive
081	Hepatocellular carcinoma	65.02	2	9	Sorafenib	4.7	HRAS	Resistant
114	Melanoma	144.4	2	9	Vemurafenib	> 10	NRAS	Resistant

### Major genomic alterations were retained in PDCs and PDXs

**Figure 4:**
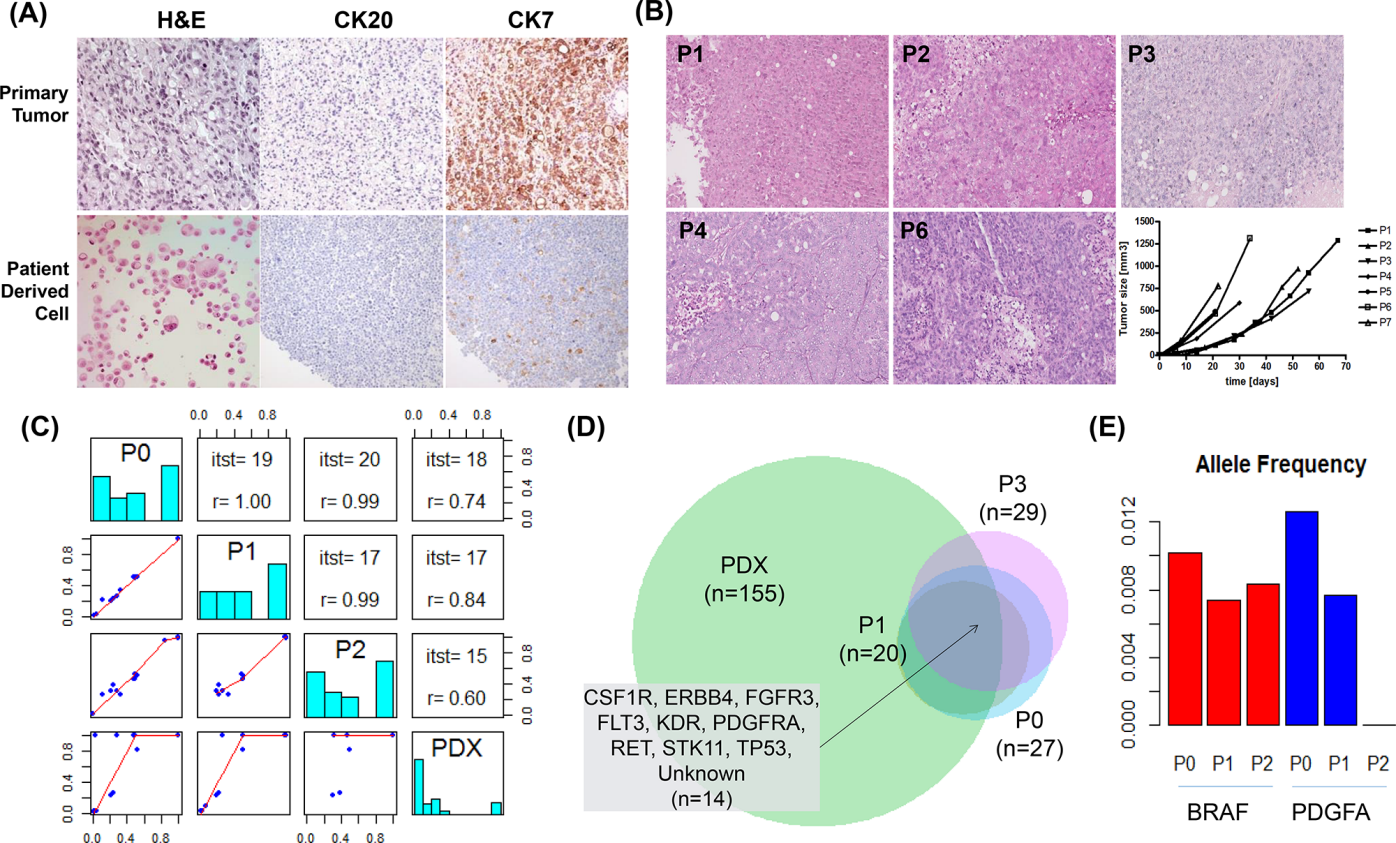
Generation and validation of patient-derived cell (PDC) models **A.** Micrographs of tissue sections and immunohistochemical analysis of PDCs and their corresponding primary tumors (40×). **B.** Comparison of PDC xenografts according to passage number. **C.** Paired comparisons of four samples. The left bottom panel shows dot plots of allele frequencies for two samples. The diagonal line shows the allele frequency histogram for four samples. The right top panel shows the number of intersections and allele frequency correlations of variants. **D.** Venn diagram of the identified variants and intersection of genes from the four samples. **E.** BRAF (red) and PDGFA (blue) allele frequency in P0, P1, and P2.

As a proof-of-concept study, we characterized ascite-derived PDCs from a patient with *RAS*-amplified GC. Primary tumor cells and PDCs generated from ascites maintained histologic features in H&E, CK7, and CK20 staining (Figure 4A). We implanted these PDCs in NSG mice to evaluate whether PDCs could be converted to a PDX model. This PDC-PDX model exhibited histological features and IHC findings similar to those of the primary tumor (Figure 4B). To confirm the genomic features of various tumor cells, we compared variant allele frequency results of the primary tumor (P0), P1 cells, P2 cells, and PDXs derived from P2 cells (Supplementary Table 3). Three samples (P0, P1, and P2) were highly correlated (0.99–1), and the numbers of identified variants were similar (from 20 to 27). PDXs showed many variants (over 100), and low correlation was observed with the other samples (0.6–0.8; Figure 4C). All 14 positions shown in Figure 4D from the intersection of four samples were known positions of dbSNPs, and the mean allele frequency was 0.77. The number of intersection variants from P0 to P2 was 17. *CDKN2B*, *PTEN1*, and *SMARCB1* were also included with genes of 14 variants. *BRAF*, *PDGFA*, and *ZHX2* were detected in only P0. A dbSNP (rs55932048) was detected in *ZHX2*, with a frequency of 0.6. *BRAF* harbored an intronic variant (chr7. hg19:g.140481514A>G) discovered in COSMIC (stomach = 11, large intestine = 6953), and *PDGFA* possessed a missense mutation (p.I670V) in COSMIC (stomach = 32, large intestine = 26). As presented in Figure 4E, variants of BRAF and PDGFA had low allele frequencies (approximately 0.15), making them difficult to identify. Additionally, when investigating the position status of BRAF and PDGFA for all samples, variant allele frequencies seemed to be lower in P2 than in P0 (Figure 4E). For instance, PDGFA p.I670V, which was present in P0, was not identified in P2. Moreover, variants in *BRAF* and *PDGFA* were not identified in PDXs.

### Comparison of gene expression between GC PDC and the TCGA/ACRG cohort

**Figure 5:**
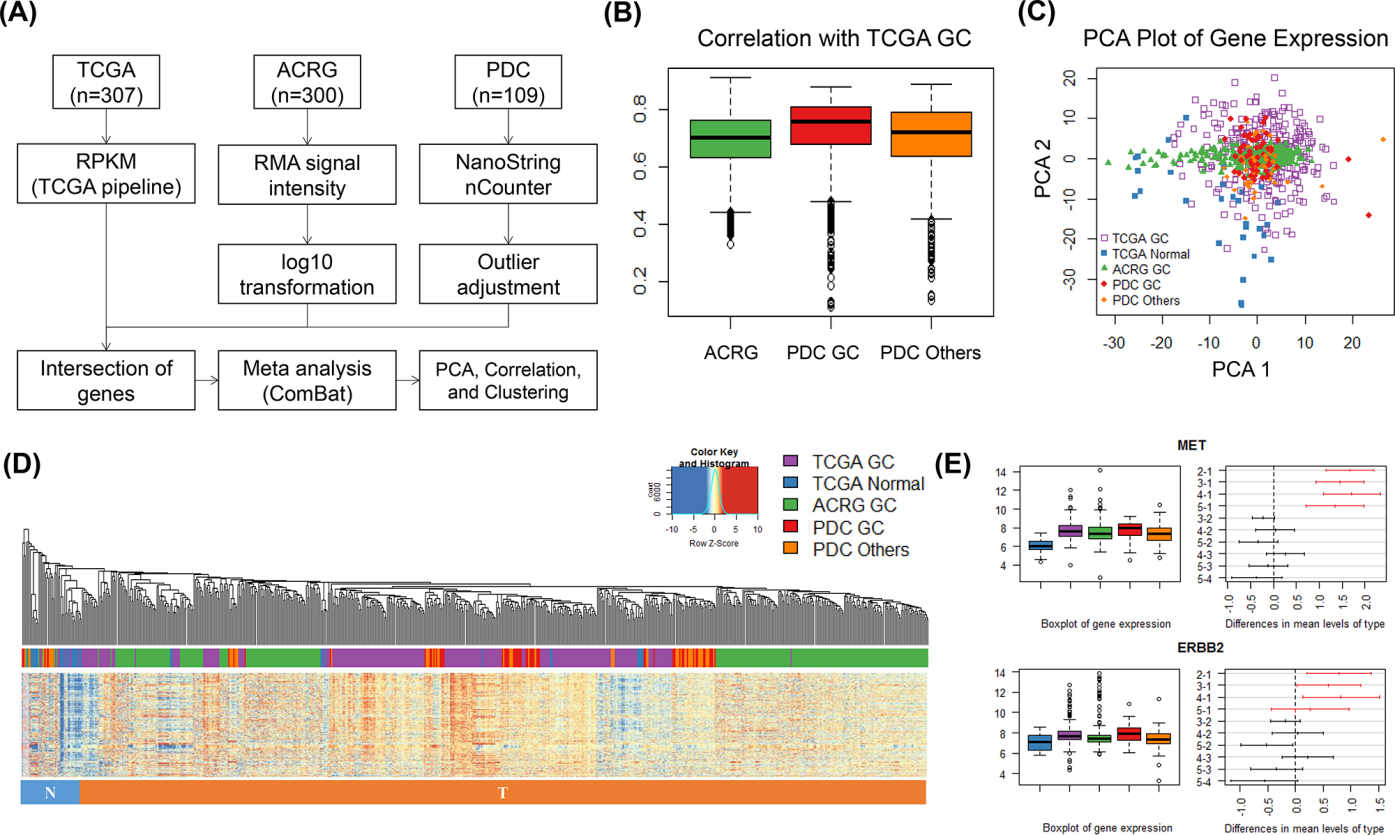
Gene expression analysis of multiple gastric cancer cohorts and PDCs **A.** Gene expression analysis work flow for integration of three different datasets. **B.** All-pair sample correlations of TCGA GC samples with ACRG, PDC PC, and PDC others (samples excluding gastric cancer). **C.** Two-dimensional plot of three dataset samples using principal component analysis. Samples including TCGA GC, TCGA normal, ACRG, PDC GC, and PDC others are indicated in the key. **D.** Sample hierarchical clustering of gene expression (485 genes and 716 samples) after meta-analysis. The color of the bottom bar indicates the sample type: blue, cluster-enriched normal samples; red, cluster-enriched tumor samples. **E.** Case studies of the differential expression of oncogenes (i.e., *MET* and *ERBB2*). Bar plots show the expression levels of these genes in five different groups. Differences were plotted by HSD tests among five groups with 95% family-wise confidence level. Red indicates the comparison of normal and tumor samples, and black indicates comparisons between tumor samples.

In order to investigate the relevance of changes in gene expression in PDCs, we integrated three different datasets (TCGA GC, Asian Cancer Research Group [ACRG] GC, and our PDC cohort) [19, 20]. ACRG and GC PDC samples were from Korean patients, while samples in the TCGA dataset were collected from multiple ethnic groups. TCGA was the only dataset to include normal samples (*N* = 33). For cross-platform gene expression comparison, meta-analysis was performed using the workflow shown in Figure 5A. Between-tumor cohort similarity was inferred from sample correlations between different cohorts. Correlation distributions are shown in Figure 5B. All sample correlations between cohorts were quite similar. The mean correlation between ACRG and TCGA was 0.69, while that between ACRG and PDC GC was 0.73 and that between ACRG and PDC except GC was 0.7. Heterogeneity of GC was present in TCGA GC samples (Figure 5C). PDC GC samples were clustered well with both TCGA GC and ACRG GC cohorts (Figure 5D). Korean patient samples of ACRG and PDC GC were centralized in the PDC plot core and seemed to be less diverse than the TCGA cohort. When investigating differential expression of four major known oncogenes in GC, we found that *MET* (*p* = 0.00) and ERBB2 (*p* = 0.00) were differentially expressed between normal and tumor tissues (Figure 5E). Taken together, gene expression profiling of the GC PDC cohort correlated well with the large genome cohorts TCGA and ACRG.

## DISCUSSION

Accurate prediction of antitumor efficacy for targeted agents before clinical trial design and implementation in patients with metastatic cancer is essential to improve treatment outcomes. Ideal preclinical models should closely resemble actual tumors in terms of molecular profiles and clinical behaviors. In this study, we developed a PDC model that exhibited histological features, genomic profiles, and functional behaviors similar to those of real tumors in patients with metastatic cancer. To the best of our knowledge, this is the first study to demonstrate a high success rate of PDC establishment from a large number of samples from patients with cancer. In addition, we showed that the most useful source of PDCs was malignant ascites. The median time from specimen collection to P1 was 3 weeks (range, 0.5–4 weeks), and that from P1 to P2 was 2.5 weeks (range, 0.5–5 weeks). The targeted sequencing results of paired primary tumor/PDCs were highly concordant. Thus, these data have important implications in the development of appropriate model systems for drug discovery and screening.

We profiled the genomes of 116 PDCs using next-generation sequencing (NGS), targeted gene expression profiling, and nCounter CNV assays. Given the current widespread use of NGS to identify rare genetic aberrations that can also be targeted with specific drugs, there is a great need to increase the availability to preclinical models for genomic profiling. Several molecules that directly target cancer driver genes have been shown to have unprecedented activities in preclinical studies; however, many compounds fall short of their expectations once tested in clinical trials [6]. To improve the relevance of preclinical models, several factors must be considered, including faithful reproduction of the biological features and clinical courses of patients' primary tumors, the ability to rapidly establish a disease model, affordability, and simplified handling methods. While conventional cell lines are convenient and easy to use, they often have poor predictive power [22]. Although PDX models retain important biological properties that are also observed in primary tumors, these models are time consuming and difficult to generate [23, 24]. Furthermore, PDX models are limited by their cost, labor requirements, and ethical issues concerning research using animals [25]. Considering these limitations, we attempted to demonstrate the advantages of our PDC model in this study. First, we found that PDC models faithfully recapitulated primary patient tumors, retaining the molecular and gross phenotypic characteristics of the primary tumor. Second, the median time from specimen collection to PDC P1 was only 3 weeks, which is more feasible for clinical application than PDX models, which take more than 3–4 months. Third, the success rate was very high (>70% of attempted cases), and the maximum passage number was 10. Fourth, although tested in selected cases only, we demonstrated that PDCs could be successfully engrafted into immunocompromised mice; thus, PDCs can be used both *in vitro* and as a cell source for further *in vivo* analyses. The variant calling allele frequencies of P0–P2 were concordant; however, PDXs varied more from the primary tumor in terms of genomics and were less concordant with the primary tumor when compared with the concordance rates of P0–P2 cells. Thus, these data supported that PDC lines may be a valid alternative model to PDXs to test the efficacy of preclinical compounds for specific molecular targets. However, more extensive study using conversion of PDCs to PDXs should be performed to assess the accurate take-rate of cultured PDCs in animal models. In addition, direct comparison experiments of antitumor efficacy of molecularly targeted agents in PDCs versus PDXs are currently underway.

As an exploratory analysis, we tested whether the drug response profiles of PDCs were concordant with the actual clinical responses to targeted agents in selected cases. Based on our previous work, genomically characterized PDCs can be used as a useful tool to demonstrate antitumor efficacy of specific targeted agents, such as CCNE1-amplified (+) PDCs [26], HER2 (+) MET (+) GC PDCs [27], and MerTK (+) GC PDCs [28]. In this study, we demonstrated that GC PDCs derived from a patient with lapatinib-sensitive cancer were sensitive to lapatinib *in vitro*. Similarly, PDCs generated from pleural fluid in a patient with vemurafenib resistance were resistant to vemurafenib *in vitro*. We plan to generate more extensive data integrating genomic, PDC-sensitivity, and clinical outcome results. This integrative genome/PDC sensitivity/clinical outcome mapping may greatly enhance our knowledge to better predict clinical outcomes at the preclinical stage.

There are several limitations to the current study. Because PDC models do not reflect all aspects of the tumor environment, such as the presence of immunocytes and mesenchymal cells, this model may not reflect the actual treatment scenario in patients who are treated with a specific agent [29, 30]. In order to overcome this pitfall, we are currently modifying the two-dimensional PDC culture method to three-dimensional organoid cultures, with or without matrices.

In conclusion, we described a PDC model that could be applied as a useful tool for the identification of rational therapeutic strategies to be tested in clinical trials. This model is expected to aid in the discovery of additional therapeutic approaches and the identification of biomarkers in response to therapy.

## SUPPLEMENTARY TABLES









## References

[1] Shoemaker RH (2006). The NCI60 human tumour cell line anticancer drug screen. Nat Rev Cancer.

[2] Roschke AV, Tonon G, Gehlhaus KS, McTyre N, Bussey KJ, Lababidi S, Scudiero DA, Weinstein JN, Kirsch IR (2003). Karyotypic complexity of the NCI-60 drug-screening panel. Cancer Res.

[3] Gillet JP, Calcagno AM, Varma S, Marino M, Green LJ, Vora MI, Patel C, Orina JN, Eliseeva TA, Singal V, Padmanabhan R, Davidson B, Ganapathi R (2011). Redefining the relevance of established cancer cell lines to the study of mechanisms of clinical anti-cancer drug resistance. Proc Natl Acad Sci U S A.

[4] Bhowmick NA, Neilson EG, Moses HL (2004). Stromal fibroblasts in cancer initiation and progression. Nature.

[5] Ostman A, Augsten M (2009). Cancer-associated fibroblasts and tumor growth—bystanders turning into key players. Curr Opin Genet Dev.

[6] Johnson JI, Decker S, Zaharevitz D, Rubinstein LV, Venditti JM, Schepartz S, Kalyandrug S, Christian M, Arbuck S, Hollingshead M, Sausville EA (2001). Relationships between drug activity in NCI preclinical *in vitro* and *in vivo* models and early clinical trials. Br J Cancer.

[7] Daniel VC, Marchionni L, Hierman JS, Rhodes JT, Devereux WL, Rudin CM, Yung R, Parmigiani G, Dorsch M, Peacock CD, Watkins DN (2009). A primary xenograft model of small-cell lung cancer reveals irreversible changes in gene expression imposed by culture *in vitro*. Cancer Res.

[8] DeRose YS, Wang G, Lin YC, Bernard PS, Buys SS, Ebbert MT, Factor R, Matsen C, Milash BA, Nelson E, Neumayer L, Randall RL, Stijleman IJ (2011). Tumor grafts derived from women with breast cancer authentically reflect tumor pathology, growth, metastasis and disease outcomes. Nat Med.

[9] Reyal F, Guyader C, Decraene C, Lucchesi C, Auger N, Assayag F, De Plater L, Gentien D, Poupon MF, Cottu P, De Cremoux P, Gestraud P, Vincent-Salomon A (2012). Molecular profiling of patient-derived breast cancer xenografts. Breast Cancer Res.

[10] Jin K, Teng L, Shen Y, He K, Xu Z, Li G (2010). Patient-derived human tumour tissue xenografts in immunodeficient mice: a systematic review. Clin Transl Oncol.

[11] Richmond A, Su Y (2008). Mouse xenograft models vs GEM models for human cancer therapeutics. Dis Model Mech.

[12] Hidalgo M, Amant F, Biankin AV, Budinska E, Byrne AT, Caldas C, Clarke RB, de Jong S, Jonkers J, Maelandsmo GM, Roman-Roman S, Seoane J, Trusolino L (2014). Patient-derived xenograft models: an emerging platform for translational cancer research. Cancer Discov.

[13] Junttila MR, de Sauvage FJ (2013). Influence of tumour micro-environment heterogeneity on therapeutic response. Nature.

[14] Mitra A, Mishra L, Li S (2013). Technologies for deriving primary tumor cells for use in personalized cancer therapy. Trends Biotechnol.

[15] Kim S, Lee J, Hong ME, Do IG, Kang SY, Ha SY, Kim ST, Park SH, Kang WK, Choi MG, Lee JH, Sohn TS, Bae JM (2014). High-throughput sequencing and copy number variation detection using formalin fixed embedded tissue in metastatic gastric cancer. PLoS One.

[16] Koboldt DC, Zhang Q, Larson DE, Shen D, McLellan MD, Lin L, Miller CA, Mardis ER, Ding L, Wilson RK (2012). VarScan 2: somatic mutation and copy number alteration discovery in cancer by exome sequencing. Genome Res.

[17] Ramos AH, Lichtenstein L, Gupta M, Lawrence MS, Pugh TJ, Saksena G, Meyerson M, Getz G (2015). Oncotator: cancer variant annotation tool. Hum Mutat.

[18] Geiss GK, Bumgarner RE, Birditt B, Dahl T, Dowidar N, Dunaway DL, Fell HP, Ferree S, George RD, Grogan T, James JJ, Maysuria M, Mitton JD (2008). Direct multiplexed measurement of gene expression with color-coded probe pairs. Nat Biotechnol.

[19] Cancer Genome Research N (2014). Comprehensive molecular characterization of gastric adenocarcinoma. Nature.

[20] Cristescu R, Lee J, Nebozhyn M, Kim KM, Ting JC, Wong SS, Liu J, Yue YG, Wang J, Yu K, Ye XS, Do IG, Liu S (2015). Molecular analysis of gastric cancer identifies subtypes associated with distinct clinical outcomes. Nat Med.

[21] Chen C, Grennan K, Badner J, Zhang D, Gershon E, Jin L, Liu C (2011). Removing batch effects in analysis of expression microarray data: an evaluation of six batch adjustment methods. PLoS One.

[22] Voskoglou-Nomikos T, Pater JL, Seymour L (2003). Clinical predictive value of the *in vitro* cell line, human xenograft, and mouse allograft preclinical cancer models. Clin Cancer Res.

[23] Fleming JM, Miller TC, Meyer MJ, Ginsburg E, Vonderhaar BK (2010). Local regulation of human breast xenograft models. J Cell Physiol.

[24] Siolas D, Hannon GJ (2013). Patient-derived tumor xenografts: transforming clinical samples into mouse models. Cancer Res.

[25] Kelland LR (2004). Of mice and men: values and liabilities of the athymic nude mouse model in anticancer drug development. Eur J Cancer.

[26] Kim J, Fox C, Peng S, Pusung M, Pectasides E, Matthee E, Hong YS, Do IG, Jang J, Thorner AR, Van Hummelen P, Rustgi AK, Wong KK (2014). Preexisting oncogenic events impact trastuzumab sensitivity in ERBB2-amplified gastroesophageal adenocarcinoma. J Clin Invest.

[27] Ha SY, Lee J, Jang J, Hong JY, Do IG, Park SH, Park JO, Choi MG, Sohn TS, Bae JM, Kim S, Kim M, Kim S (2015). HER2-positive gastric cancer with concomitant MET and/or EGFR overexpression: a distinct subset of patients for dual inhibition therapy. Int J Cancer.

[28] Yi JH, Jang J, Cho J, Do IG, Hong M, Kim ST, Kim KM, Lee S, Park SH, Park JO, Park YS, Kang WK, Lim HY (2015). MerTK is a novel therapeutic target in gastric cancer. Oncotarget.

[29] Kim JB (2005). Three-dimensional tissue culture models in cancer biology. Semin Cancer Biol.

[30] Yamada KM, Cukierman E (2007). Modeling tissue morphogenesis and cancer in 3D. Cell.

