# Supplementary material for: Patient-derived cell models as preclinical tools for genome-directed targeted therapy
Source: Oncotarget. 2015 Jul 16;6(28):25619–30. doi: 10.18632/oncotarget.4627 (PMC4694854; doi:10.18632/oncotarget.4627)
Supplement: Supplementary file 1 [file oncotarget-06-25619-s001.pdf]

SUPPLEMENTARY TABLES

Supplementary Table S1. Physiological characteristics and growth properties of established PDCs.

Supplementary Table S2. List of somatic mutations examined by Ion AmpliSeq Cancer Panel v2.

Supplementary Table S3. Variant calling result of P0, P1, P2 and PDX. The table supports MAF format of TCGA
